# Millets, pulses, and oil seeds‐based flatbread premix: A protein‐rich functional food for healthier dietary habits and prevention of lifestyle disorders

**DOI:** 10.1111/1750-3841.70209

**Published:** 2025-04-24

**Authors:** Shraddha A. Bhoir, Deepak Sharma, Sahayog Jamdar

**Affiliations:** ^1^ Food Technology Division Bhabha Atomic Research Centre, Trombay Mumbai India; ^2^ Radiation Biology and Health Science Division Bhabha Atomic Research Centre, Trombay Mumbai India; ^3^ Homi Bhabha National Institute, Anushaktinagar Mumbai India

**Keywords:** functional foods, immunomodulation, millets and pulses, nutrition and health

## Abstract

**Abstract:**

With an increasing focus on healthier dietary alternatives, this study developed a protein‐rich, ready‐to‐cook flatbread premix using millet, pulse, and oilseed flours. The premix offers a balanced nutritional profile, providing high protein content (∼21%) and delivering 423 kcal/100 g. It demonstrated phenolic content of 0.98 mg catechin equivalents/gram of premix, which increased to 1.90 mg catechin equivalents/gram of cooked (lyophilized) product.

Antioxidant activity was evaluated through DPPH radical scavenging, yielding values of 5.28 nmol Trolox equivalents per gram for the premix and 4.82 nmol Trolox equivalents per gram for the cooked product. Similarly, hydroxyl radical scavenging activity was measured at 4.38 µmol Trolox equivalents per gram for the premix and 4.36 µmol Trolox equivalents per gram for the cooked product. The product was highly acceptable in sensory evaluations, indicating strong potential for consumer adoption. In vivo studies with Balb/c mice revealed no adverse effects on oxidative stress levels or immune function. The flatbread promoted a healthy gut microbiota composition, supporting gut health while maintaining cellular oxidative balance. Hematological analyses showed improved white blood cell and lymphocyte counts, reflecting enhanced immune resilience.

These findings highlight the premix as a promising dietary alternative to traditional carbohydrate‐rich staples. With its nutrient density and functional benefits, the flatbread premix offers a practical solution for improving dietary habits and addressing lifestyle‐related health concerns. This study underscores the importance of incorporating such functional foods into daily diets to promote better health and prevent nutrition‐related disorders. Further research is warranted to explore its broader health impacts over extended durations.

**Practical Application:**

The ready‐to‐cook flatbread premix made from millets, pulses, and oilseeds offers a simple and nutritious way to enhance daily diets. Rich in protein and supplemented with various oilseeds as a source of polyunsaturated fatty acids, it provides a healthier alternative to regular flatbreads. It is convenient and ideal for maintaining a balanced diet and also supports the management of lifestyle‐related health issues such as obesity. With its antioxidant properties and ability to support a healthy gut microbiota, this flatbread contributes to overall health. It promotes better eating habits without compromising taste or convenience.

## 1 INTRODUCTION

Diet plays a pivotal role in shaping human health and well‐being (Al Mutairi et al., 2022; Gheonea et al., 2023; Shridhar et al., 2015). In recent decades, there has been a significant global shift in dietary patterns, with increasing consumption of calorie‐dense, nutrient‐poor foods high in refined sugars, unhealthy fats, and excess salt. These dietary habits are closely linked to the rising prevalence of lifestyle‐related diseases such as obesity, type 2 diabetes, cardiovascular disorders, and certain cancers. This alarming trend has fueled interest in developing functional foods—nutrient‐rich dietary options that not only provide basic nutrition but also offer health benefits capable of preventing or mitigating chronic conditions (Bhardwaj et al., 2016; Medina‐Remón et al., 2018; Monda et al., 2024; Onyeaka et al., 2023; Willcox et al., 2009).

The Food and Agriculture Organization (FAO) of the United Nations defines functional foods as foodstuffs that provide health benefits beyond basic nutrition, demonstrating specific health or medical advantages, including the prevention and treatment of disease. These foods are often enriched with bioactive compounds such as antioxidants, polyphenols, and dietary fiber, which are known to enhance physiological functions and reduce disease risk (Banwo et al., 2021; Dixit et al., 2023). The development of such foods has gained momentum as they align with the dual goals of promoting healthier lifestyles and addressing widespread nutritional deficiencies in modern diets.

Traditional crops such as millets, pulses, and oilseeds have emerged as promising candidates for functional food development due to their nutrient density, bioactive potential, and environmental sustainability. These crops are particularly rich in proteins, essential amino acids, dietary fiber, vitamins, minerals, and phytochemicals with antioxidant properties, making them ideal ingredients for functional food formulations (Amadou, 2022; Tripathi et al., 2021). Additionally, whey protein isolate has proven to be a versatile ingredient in functional food development. As a complete protein with high biological value and digestibility, whey protein is especially effective when combined with plant‐based proteins, complementing their amino acid profiles to create nutritionally balanced food products. Flax seeds, chia seeds, and pumpkin seeds are widely recognized for their numerous health benefits, including their anti‐inflammatory effects. Flax seeds and chia seeds are known to be rich in omega‐3 fatty acids and antioxidants. Flax seeds have been shown to reduce inflammation and support overall health (Parikh et al., 2019) while chia seeds have been associated with decreased inflammation and improved cardiovascular health (Pam et al., 2024). Additionally, pumpkin seeds have been found to contain phytochemicals, such as phytosterols and flavonoids, that may help alleviate inflammation and lower the risk of chronic diseases (Batool et al., 2022). Regular consumption of these seeds is suggested to contribute to better health by providing essential nutrients and reducing inflammation. The fusion of plant proteins from millets, pulses, and oilseeds with whey protein isolate offers a unique opportunity to develop innovative, high‐protein foods that meet modern nutritional needs (Patel, 2015).

The present study introduces a novel functional food premix formulated from millets, pulses, oilseeds, and whey protein isolate. Designed for easy preparation as a flatbread, the premix contains approximately 30% protein and integrates the nutritional benefits of both plant‐ and dairy‐based ingredients. Unlike conventional dietary interventions that aim to counteract the negative effects of unhealthy diets, this functional food is designed to provide a healthier and more wholesome dietary alternative that promotes long‐term health. By incorporating bioactive compounds with antioxidant properties, such as phenolics, this premix aligns with dietary approaches aimed at preventing oxidative stress, a key factor in the onset of lifestyle‐related diseases. Furthermore, the premix supports better dietary habits by offering a convenient and nutritionally superior alternative to processed and refined foods. Its balanced nutrient profile—featuring proteins, dietary fiber, and essential micronutrients—enhances overall dietary quality.

The use of traditional crops like millet and pulses also aligns with global sustainability goals, as these crops are drought‐resistant and environmentally friendly compared to conventional cereals. This study highlights the formulation and characterization of the premix, emphasizing its potential role as a functional food for improving dietary patterns. By focusing on nutrient density, bioactive functionality, and convenience, this work contributes to the growing body of evidence supporting the importance of functional foods in addressing lifestyle‐related diseases.

## 2 MATERIAL AND METHODS

Whey protein isolate (Profoods Nutrition Pvt. Ltd., Mumbai, Maharashtra), flours used in the premix namely; green gram (*Vigna radiata*), chickpea (*Cicer arietinum*), jowar (*Sorghum vulgare*), and bajra (*Pennisetum glaucum*) flours; seeds of flax (*Linum usitatissimum*), pumpkin (*Cucurbita pepo*), chia (*Salvia hispanica*); and spices like turmeric (*Curcuma longa*), chilli (*Capsicum annuum*) powder, cumin (*Cuminum cyminum*) powder, coriander (*Coriandrum sativum*) and salt were obtained from local market. All the reagents were analytical grade from Himedia Laboratories, India.

### 2.1 Preparation of premix

**FIGURE 1:**
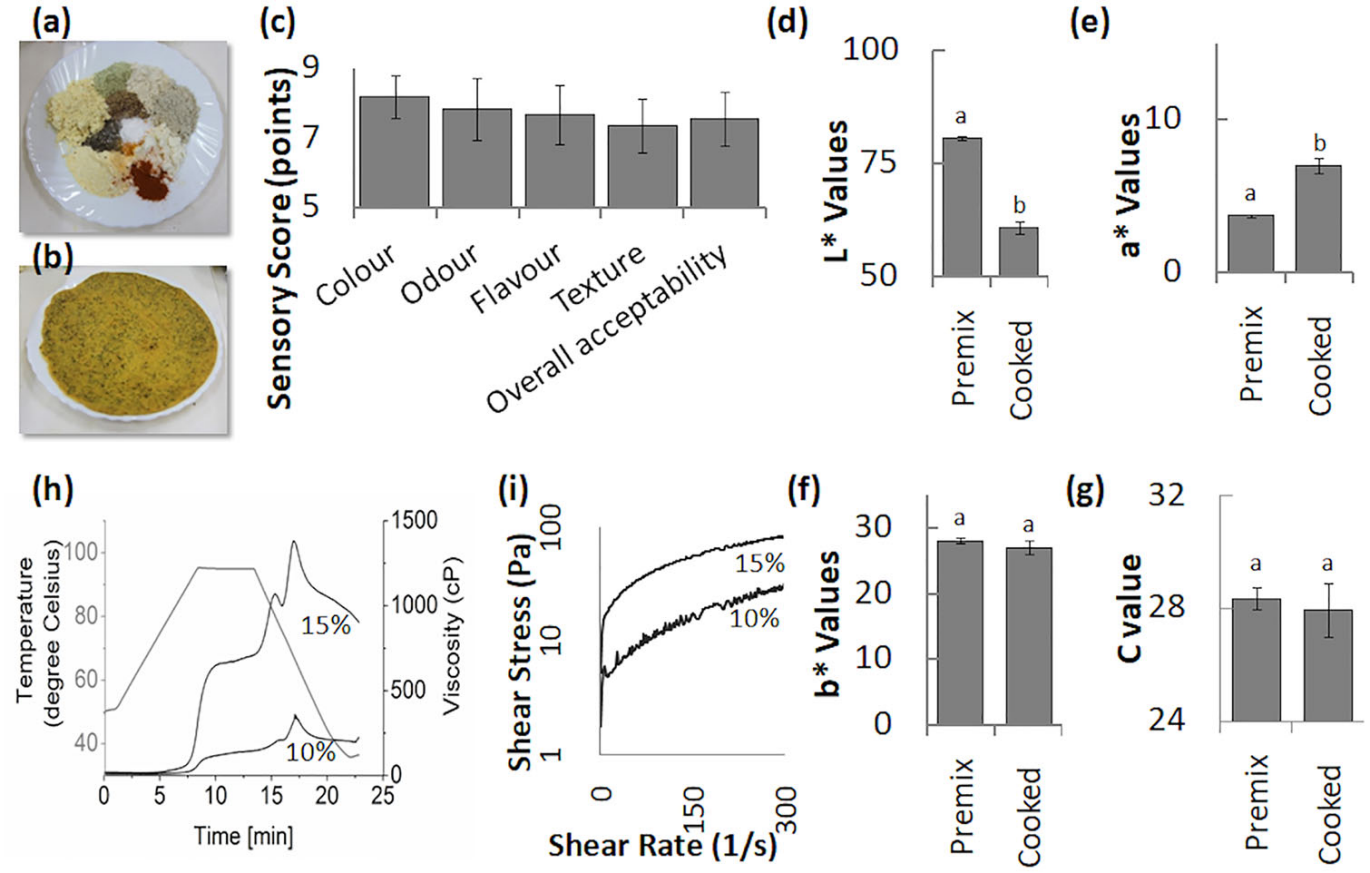
Visual representation of flatbread composition and quality. (a) Components of flatbread premix. (b) Flatbread: post‐cooking. (c) Sensory score of flatbread quantifying the qualitative attributes. (d–g) Color expressed in CIELAB color space. (d) A quantitative display of lightness values (L*), (e) redness or greenness axis (a*) values, (f) yellowness or blueness (b*) values, (g) chroma (C*) values, reflecting the flatbread's color purity and intensity, (h) pasting properties and (i) flow behavior at 10% and 15% concentration of premix homogenate. The same lower letter within a group denotes no statistically significant difference in the mean of the samples due to pre‐treatment for flour.

The flat bread premix was prepared by mixing all the flours, whey protein isolate, powdered seeds and spices and salt in the proportion mentioned in Table 1 and Figure 1a. All the seeds were powdered before use. The premixes were stored in polypropylene bags at 4°C till further use.

**TABLE 1:** Composition of flatbread premix.

Component	Percent	Component	Percent	Component	Percent
Whey protein isolate	11.0	Flax seeds	5.5	Cumin	1.4
Moong flour	21.5	Pumpkin seeds	5.5	Chili powder	1.4
Chickpea flour	21.5	Chia seeds	5.5	Coriander powder	1.4
Sorghum flour	11.0	Turmeric	0.3		
Pearl millet flour	11.0	Salt	2.9		

The formulation of the flatbread premix was designed to achieve a target protein content of 25%–30%, in alignment with the nutritional objective of creating a high‐protein functional food. The known protein contents of individual ingredients were used as reference data. Published nutritional data guided the calculation of their contributions to the total protein content of the mix. Ingredients were combined in varying proportions to ensure that the theoretical protein content of the final premix approached the target range. These calculations took into account not only the protein content of each ingredient but also their functional and sensory roles in flatbread preparation, such as texture, taste, and binding properties. The protein content of the optimized premix was analytically validated using the Kjeldahl method and found to be 22%, which was close to the theoretical target.

The composition of market samples mentioned in Table S1 was obtained from the labeling on their packets.

### 2.2 Preparation of flat‐bread from the premix, nutritional analysis, and estimation of color

Premix (80 g) was mixed with 130 g of water and mixed thoroughly to obtain a slurry, which was poured and spread on the pre‐heated griddle. The bread was roasted for 2 min on both sides. Moisture content was determined gravimetrically. Fat and protein content was determined using methods FSSAI 03.039:2022 and FSSAI 03.016:2022, respectively. Carbohydrate content was estimated by the difference method. Vitamins were estimated by HPLC. The color of the samples was determined with a Konica Minolta colorimeter (Chiyoda City, Tokyo, Japan) using the CIE lab color parameters. The color was expressed as Hunter's *L*, *a*, *b*, values. The nutritional values of market samples mentioned in Table S1 were obtained from the labeling on their packets.

### 2.3 Estimation of pasting properties and flow behavior of the flatbread premix

Pasting properties and flow behavior of the flatbread premix were obtained using a Starch cell (ST24) using Anton‐Paar rheometer (Model No. MCR 102e, Austria) as described by Mallikarjunan et al. (2024). Suspensions of varying concentrations were prepared by dispersing the premix in 20 g of distilled water. The sample was maintained at 50°C for 5 min with pre‐sheer rotation at 960 rpm before performing the measurements. The test was performed with viscosity being measured under a rotation of 160 rpm. The sample suspension was maintained at 50°C for 1 min, then heated to 95°C in 8.5 min and held at this temperature for 5 min, followed by cooling to 37°C in the next 18 min. The sample was then held at 37°C for 1 min. The pasting temperature (the temperature where viscosity first increases by at least 25 cp over a 20 s period), peak time (the time at which peak viscosity occurred), peak viscosity (the maximum hot paste viscosity), final viscosity (the viscosity at the end of test after cooling to 37°C and holding at this temperature) were calculated from the pasting curve (Ragaee and Abdel‐Aal, 2006). Following the estimation of pasting properties, same sample suspension was subjected to measurement of shear rate, shear stress, and viscosity to estimate flow behavior post‐gelatinization.

### 2.4 In vitro antioxidant activity

To perform the assays, the flatbread premixes were either used uncooked or cooked in the form of flatbread, freeze‐dried, and powdered. Samples (10 g) were extracted with 25 mL of 50% ethanol for 1 h at 37°C and the extract was used for analysis.

#### 2.4.1 Total phenolics content of flatbread

Total phenolic content was estimated using the Folin–Ciocalteu reagent (Singleton and Rossi, 1965). Briefly, 100 µL of appropriately diluted extract was mixed with 750 µL of 0.2 N FC reagent and incubated at room temperature (RT) for 5 min. 750 µL of 6% NaHCO_3_ was added, and absorbance was read at 725 nm using UV–visible spectrophotometer (Jasco Corp, Hachioji‐shi, Tokyo Japan) after 90 min of incubation at RT. The total phenolic content was expressed as µg gallic acid equivalent (GAE)/g sample (Zilani et al., 2017).

#### 2.4.2 K‐ferricynanide reducing power

To determine the reducing power, 500 µL of appropriately diluted extract was mixed with 500 µL of Na‐phosphate buffer (200 mM, pH 6.6) and 500 µL of 1% K‐ferricyanide and incubated for 20 min in a water bath at 50°C. Trichloroacetic acid (TCA, 10%, 500 µL), 2 mL of distilled water, and 400 µL of 0.1% FeCl_3_ were added to the reaction mixture, and the absorbance was read at 700 nm. 1 unit of absorbance at 700 nm corresponded to a reduction of 0.5 µmoles of K‐ferricyanide. The reducing power was expressed as µmoles of K‐ferricynanide reduced per gram of the sample (Yen and Tsai, 1993).

#### 2.4.3 DPPH radical scavenging activity

DPPH radical scavenging activity was estimated by treating an appropriately diluted extract with DPPH reagent (10 mg/100 mL ethanol) for 20 min at RT in the dark. The reduction in absorbance was read at 517 nm (Brand‐Williams et al., 1995). The percent radical scavenging activity was calculated as (*A*_B_ − *A*_S_) × 100 / *A*_B_, where *A*_B_ is the absorbance of the blank and *A*_S_ is the absorbance of the sample. The radical scavenging activity was compared with Trolox and expressed as µmoles of Trolox equivalent per g of sample.

#### 2.4.4 Hydroxyl radical scavenging activity

Similarly, hydroxyl radical scavenging activity was determined by mixing 1 mL of appropriately diluted extracts with 1 mL of phosphate buffer (0.1 M, pH 7.4) containing 1 mM Fe_3_Cl, 1 mM EDTA, 1 mM ascorbic acid, 30 mM deoxyribose, and 20 mM H_2_O_2_. After 90 min of incubation at 37°C, 2 mL of 2% TCA, and 2 mL of 0.02 M TBA were added and incubated in a boiling water bath for 15 min. The absorbance was read at 532 nm. Calculations were performed as mentioned in the previous assay.

### 2.5 Animal studies

The Institutional Animal Ethics Committee of Bhabha Atomic Research Centre, Government of India, has approved the animal studies, and the guidelines issued by the ethics committee regarding the maintenance and dissections of small animals were strictly followed. All the animals were housed in conventional cages on corncob bedding and with proper ventilation. Each cage contained 3–6 animals with the same diet regime. Feed and water were provided ad libitum. The environment was maintained on 12:12 h of light‐dark cycle with temperatures around 23°C and relative humidity of about 50% ± 10%.

#### 2.5.1 Comparison of cooked flat bread with standard chow diet

To evaluate the effect of consumption of the flatbread on physiology and general health of mice, 6 week old mice were fed with cooked flatbread for four consecutive days per week and with chow diet for remaining three days in week, to prevent deficiency of micronutrients. This feeding pattern was continued for 12 weeks. The change in weight of mice and the amount of food consumed were noted regularly. The mice were sacrificed to harvest blood and spleen. Six mice were studied per treatment.

#### 2.5.2 Comparison of cooked flatbread (CB) with high fat high sugar (HFHS) pancake

Six week old mice were either fed with CB or HFHS pancake for 32 days. The HFHS diet consisted of 30 g refined flour, 30 g sugar, 22.5 g sunflower oil, 17.5 g whey protein isolate, 2 g wheat fiber, and 60 g water, which was cooked on a griddle in the form of pancakes. The change in weight of mice and the amount of food consumed were noted regularly.

Fecal samples were collected from the cages on the 32nd day and subjected to microbiological analysis for enumeration of total viable count (aerobic and anaerobic), bifidobacterium count, and lactic acid bacteria count. The fecal samples were suspended in sterile normal saline and appropriately diluted before plating on sterile plates of plate count agar, bifidobacterium selective media and De Man–Rogosa–Sharpe agar. Blood samples were withdrawn from the live mice on 11th and 32nd day in Na‐heparin coated tubes, and complete blood count was estimated using auto haematology analyser (Rayto RT‐7600 veterinary Auto Hematology Analyzer). The serum glucose levels were determined by an enzymatic colorimetric test using a glucose oxidase‐peroxidase kit (Accucare, Labcare Diagnostics (India) Pvt. Ltd). At least nine mice were studied per treatment.

### 2.6 Statistical analysis

Data obtained from experiments were analyzed using the MS Excel 2007 (Microsoft Corp., Redmond, Washington, USA). All the experiments were performed in triplicates, and descriptive statistics, including means and standard deviations, were calculated. Differences between groups were assessed either using independent samples two‐tailed *t*‐tests or ANOVA with significance set at *p* < 0.05. All the data are presented as mean ± standard deviation.

## 3 RESULTS

### 3.1 Nutritional and sensorial analysis of the functional food

The premix developed was a free‐flowing powder which exhibited a creamish‐yellow hue, and the flatbread prepared from it was vibrant yellow, and its texture was soft but firm (Figure 1b). Analysis revealed the premix contained 8.2 g moisture, 59 g carbohydrates, 11.2 g fats, and 21.6 g proteins per 100 g, totaling 423.2 kcals per 100 g of the premix, while the cooked product contained 49.9 g moisture, 34.3 g carbohydrates, 5.2 g fats, and 10.67 g proteins per 100 g, totaling 226.7 kcals (Table 2).

**TABLE 2:** Nutritional composition of cooked product.

Component	Raw (Premix) per 100 g	Cooked (Flatbread) per 100 g
Energy (kCal)	423.2 ± 1.6	226.4 ± 1.1
Protein (g) (energy from protein)	21.6 ± 0.1 (86.4 ± 0.5 kCal, 20.4 ± 0.2% of the total energy)	10.67 ± 0.2 (42.7 ± 1.0 kCal, 18.9 ± 0.5% of total energy)
Fat (g)	11.2 ± 0.1	5.2 ± 0.0
Carbohydrate (g)	59 ± 0.2	34.2 ± 0.5
Moisture (g)	8.2 ± 0.2	49.9 ± 0.3

The functional food (FF) premix developed in this study combines a diverse blend of millets, pulses, oilseeds, and whey protein isolate to deliver superior nutritional benefits compared to four representative market samples. FF provides higher protein content (21.6 g/100 g) than any market sample, with 20.4% of its energy derived from protein, indicating a balanced macronutrient profile and a substantial improvement over market samples that provide only 8.2% to 14.9% (Table S1). This is supported by its inclusion of whey protein isolate and flours of pulses, both known for their high‐quality protein and digestibility.

In contrast, the market samples either rely heavily on carbohydrate‐rich ingredients like rice, wheat, and corn flours, and few consisted of a mix of pulses and millets. But neither of similar of kind of premixes in the market included a variety of nutrient‐dense oilseeds (Table S1). FF reported in this study incorporates nutrient‐dense oilseeds such as flax, chia, and pumpkin seeds. These ingredients provide healthy fats, essential omega‐3 fatty acids, and bioactive compounds known for their antioxidant and anti‐inflammatory properties. Additionally, its millet content ensures a low glycemic index and nutrient diversity. By integrating functional ingredients and achieving a balanced energy‐protein profile, FF stands out as a healthier, innovative alternative that addresses modern dietary challenges and promotes long‐term health. Notably, proteins in the FF contributed more than 20% of the product's energy, meeting the EU's criteria for ‘protein‐rich’ designation (Regulation (EC) No, 1924/2006).

The color of the product was expressed using the Hunter's Lab scale. The mean *L* value, representing the lightness and characterizing the flatbread's color intensity and brightness, crucial for assessing visual appeal and consumer acceptance, was observed to be 80.68 for the premix, decreasing to 60.73 after cooking. The ‘*a*’ value, representing the degree of redness or greenness, was recorded as 3.71 for the premix and increased to 7.00 in the cooked product. The ‘*b*’ value, representing blueness or yellowness, and the *C* value, representing chroma and relative saturation, remained unchanged after cooking and were noted as 28.10 and 28.53 for the premix and 27.04 and 27.93 for the cooked product, respectively.

Sensory evaluations of flatbread made from these premixes revealed consistent high acceptance scores, with no sample falling below seven points for color, odor, flavor, texture, or overall acceptability, indicating the good potential to yield high‐quality flatbread with favorable sensory attributes.

### 3.2 Pasting properties and flow behavior

The pasting properties of the functional food premix were investigated in triplicates, and their representative plots are shown in Figure 1h,i. A sharp rise in the viscosity (at least 25 cP/ 20 s) was estimated at 95.2°C and 90.6°C for premix suspensions with concentrations of 10% and 15%, respectively. This may be attributed to the gelatinization of starch contributed by the millets and pulses in the premix, and/or thermal degradation and aggregation of the proteins mainly from whey protein isolates, pulses and oilseeds. Two sharp peaks were noted at 15.567 and 17.1 min at temperatures 78.2°C and 66.5°C with viscosities 251.3 ± 62.225 and 370.2 ± 13.152 cP, respectively, for 10% premix suspension. Similar peaks were also seen in 20% premix suspension at 15.3 and 16.967 min and temperature 80.2°C and 67.4°C with viscosities 1110 ± 56.568 and 1503.5 ± 168.998 cP, respectively. It must be noted that the peak viscosity is observed during the cooling phase, indicating the complex interactions amongst the proteins, fats, and fibers that may be at interplay. Final viscosities were detected to be 231.45 ± 10.677 and 995.25 ± 132.583 cP for 10% and 15% premix suspensions, respectively. The consistency index increased with higher premix concentration, rising from 0.91 ± 0.39 at 10% to 5.42 ± 0.28 at 15% (*p* = 0.006). In contrast, the flow behavior index decreased from 0.58 ± 0.06 to 0.47 ± 0.01, respectively (*p* = 0.130).

### 3.3 Total phenolics content and the antioxidant activity

Total phenolics content, ferric reducing power and DPPH radical scavenging activity were measured to evaluate the antioxidant potential of premixes (Table 3). The phenolic compounds, assessed with Folin's reagent, were estimated to be 0.98 mg catechin eq./g premix and 1.90 mg catechin eq./g cooked product. The phenolic content of the product was comparable to other similar products found in the literature (Jiang et al., 2023; Mildner‐Szkudlarz–Jan and Szwengiel, 2010). Detectable total phenolic content notably increased post‐cooking. This increase of approximately threefold may be due to improved extractability of the phenolics. Changes in phytochemicals upon cooking may result from two opposite phenomena: thermal degradation, which reduces their concentration; and second, a matrix softening effect, which increases the extractability of phytochemicals, resulting in a higher concentration with respect to the raw material (Palermo et al., 2014). The premix exhibited a reduction of 25.00 µmoles of K‐ferricyanide reduced/g sample, and this reducing power was reduced by cooking to 22.91 µmoles of K‐ferricyanide reduced/g sample. The radical scavenging activity was unaffected by the cooking process and was detected to be equivalent to 5.28 µmoles trolox/g premix and 4.82 µmoles trolox/g cooked product for DPPH radical. Similarly, the hydroxyl radical reducing power was noted to be 4.38 and 4.36 µmoles trolox eq/g of premix and cooked sample, respectively.

**TABLE 3:** Phenolic content and in vitro antioxidant activity of the premix and the cooked flatbread.

	Raw	Cooked
Total phenolic content (mg catechin eq./g sample)	0.98 ± 0.08 a	1.90 ± 0.07 b
Reducing power (µmoles of K‐ferricyanide reduced/g sample)	25.00 ± 2.75 a	22.91 ± 1.12 b
Radical scavenging activity	DPPH (µmoles trolox eq/g sample)	
	5.28 ± 0.55 a	4.82 ± 0.39 a
	Hydroxyl (µmoles trolox eq/g sample)	
	4.38 ± 0.37 a	4.36 ± 1.19 a

*Note*: Values are expressed as mean ± standard deviation (SD) for experiments performed in triplicates. Statistical significance was determined using unpaired, two‐tailed *t*‐tests with *p* < 0.05 considered statistically significant. The same lower letter in a row denotes no statistically significant difference in the mean of the samples.

### 3.4 Animal studies

#### 3.4.1 Comparison with chow diet

**FIGURE 2:**
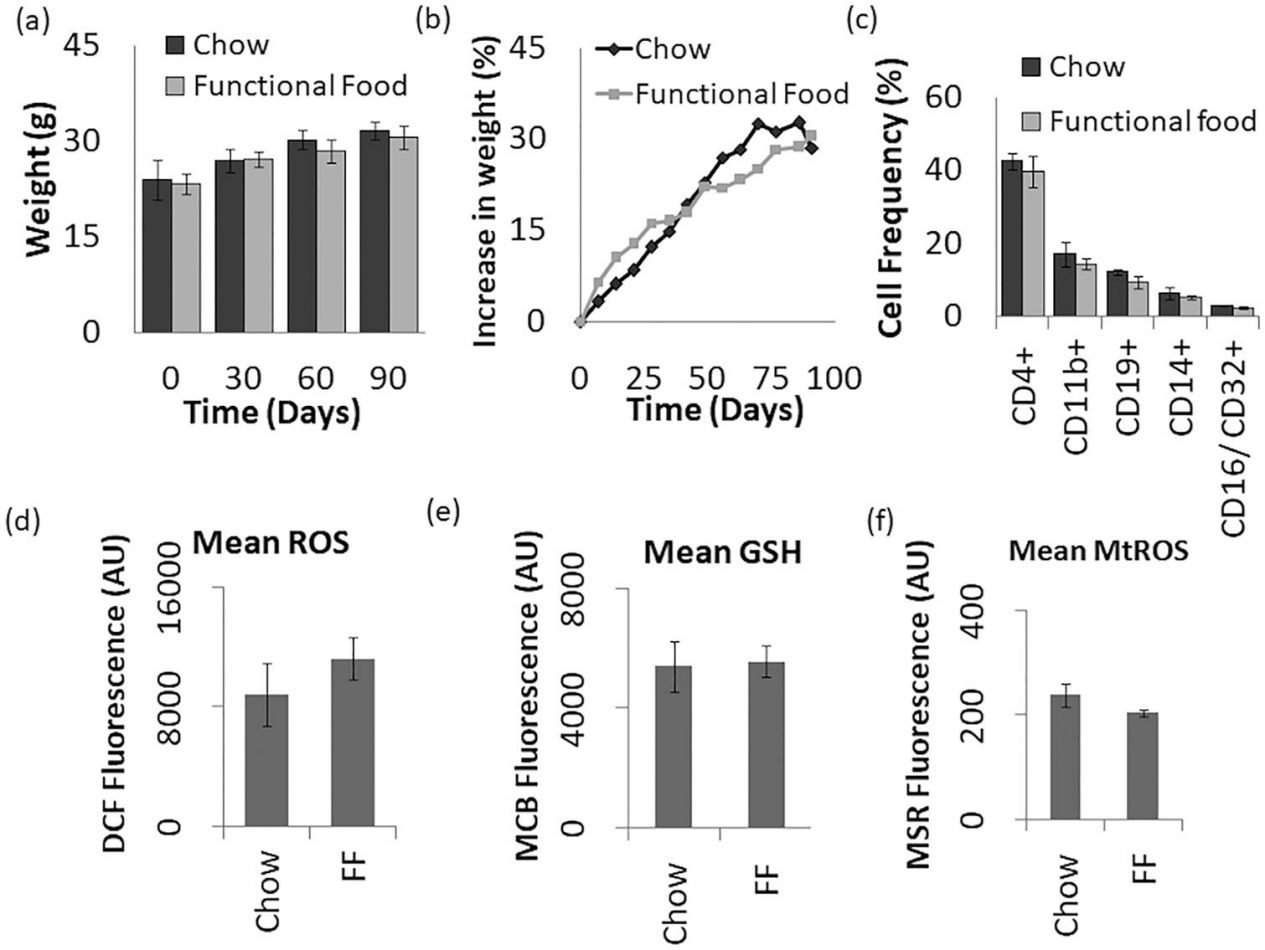
Evaluation of consumption of cooked flatbread: (a) Absolute change in weight of mice post‐consumption of cooked flatbread indicating the physiological impact on systemic homeostasis, (b) percentage change in weight of mice providing a standardized assessment of the nutritional impact. (c) Quantitative depiction of surface‐stained spleenocyte populations, delineating immune cell alterations indicative of potential immune system modulation. (d–f) Oxidative status of spleenocytes: (d) DCFDa fluorescence depicting intracellular reactive oxygen species (ROS) levels and oxidative stress status, (e) MCB fluorescence for highlighting alterations in intracellular glutathione levels, (f) MSR fluorescence for delineating mitochondrial ROS levels. Same lowercase letters indicate no statistically significant difference in the mean of the samples between mice fed the control diet and those fed the functional food.

Mice were administered the functional food for 4 days per week, complemented by a standard chow diet for the remaining 3 days, to prevent the deficiency of micronutrients, which are added specifically to the standard chow diet but not to the functional food. At the onset of the feeding trials, control mice exhibited a mean weight of 24.11 g, whereas test mice weighed in at 23.41 g. Throughout the study, mice from both groups exhibited robust health, maintained similar activity levels, and showed typical weight progression (Figure 2a,b). By the end of 3 months, control mice registered a weight of 31.78 g, reflecting a weight gain of 28.63%, while their functional food‐fed counterparts reached 30.71 g, representing a weight gain of 30.57%. Remarkably, the percentage increase in weight observed in mice consuming the functional food closely paralleled that of control mice fed a standard chow diet. Also, all the animals, irrespective of the diet groups, showed similar activity, fur quality, posture, and food intake. None of the mice, during the course of the experiment, went in isolation or exhibited hunched‐back posture or other signs of illness. These findings strongly indicate that the functional food adequately met the animals' nutritional and caloric needs without impeding their growth.

To assess the impact of functional food on the immune system of mice, spleen immune cells were quantified using surface antigen staining followed by flow cytometry Figure 2c. It is worth noting that while B‐cells (CD19+) and macrophages (CD14+) tend to remain stable during aging, dendritic cells (CD11b+) and NK cells (CD16/32+) typically increase, while T_H_‐cells (CD4+) decrease (Pinchuk et al., 2008; Scholz et al., 2013; Shaw et al., 2010). Analysis revealed a notable effect of functional food, leading to decreased proportions of CD14+ (2.67%), CD11b+ (14.39%), and CD19+ (9.42%) cells compared to chow‐fed mice (3.45%, 17.13%, and 13.68%, respectively). Conversely, no significant alterations were observed in the proportions of CD4+ cells (39.84% vs. 42.67%) and CD16/32+ cells (2.38% vs. 2.87%). Statistically, the functional food was found to lower the proportions of certain immune cells compared to chow‐fed mice.

To assess the oxidative status within the spleen cells of the mice, mean levels of DCFDA, MSR, and MCB fluorescence were estimated, serving as indicators of total cellular ROS, mitochondrial ROS, and GSH levels, respectively. As depicted in Figure 2d–f, no significant variance was observed between chow‐fed and functional food‐fed mice, indicative of a lack of adverse effects on the animals' redox status. These results strongly suggest that the consumption of functional food did not perturb the oxidative status of the mice, thus positioning it as a safe dietary formulation for preserving whole‐body redox homeostasis.

#### 3.4.2 Comparison with western diet (high‐fat, high‐sugar diet)

**FIGURE 3:**
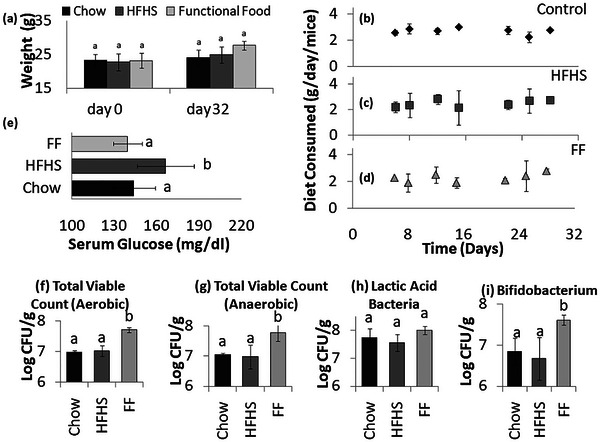
Assessment of potential anti‐aging effects of cooked flatbread consumption: (a) The absolute change in mouse weight following consumption of cooked flatbread; (b–d) amount of food consumed by control (b), functional food (c), and HFHS (d) mice groups; (e) quantitative assessment of serum glucose levels on day 32, providing insights into glycemic control and metabolic health status; (f–j) microbial abundance and potential modulation of total aerobic bacterial count (f), total anaerobic bacterial count (g), lactic acid bacteria count (h), and bifidobacterium count (j). The same lowercase letters indicate no statistically significant difference in the mean of the samples between mice fed the control diet, those fed the functional food, and those fed with HFHS diet.

The potential anti‐aging properties of functional food and its impact on the overall health and well‐being of the mice were investigated through a comparison with mice fed on high‐fat, high‐sugar (HFHS) diet. The HFHS diet was administered for 4 days per week, followed by a standard chow diet for the remaining 3 days. Over the course of 32 days of administering diet to the mice, the weights increased by 4% (24.1 g) in the chow‐fed group, 9% (24.9 g) in functional food‐fed mice and 11% (27.7 g) in HFHS‐fed mice group (Figure 3a). However, there was no statistically significant difference in the weights of mice within the groups by the end of the experiment. Figure 3b–d also illustrates the daily food consumption per mouse. On average, mice consumed 2.5 to 3.0 g of chow diet and 2.0 to 2.8 g of functional food or HFHS diet per day, indicating no significant variance in food intake.

On day 32, the glucose level in the serum of chow‐fed group (136.6 ± 22.6 mg/dL) was found to be comparable to that in the serum of functional food‐fed mice (135.7 ± 14.7 mg/dL). Notably, the glucose levels in the serum of HFHS‐fed mice group were found to be significantly higher at 166.3 ± 20.1 (Figure 2e).

The impact of functional food consumption on fecal microbiota composition is represented in Figure 3f–i. Microbiological analysis of fecal samples revealed significant variations in microbial populations among the different dietary groups. The total viable count of aerobic bacteria was elevated in the functional food fed mice group (7.70 ± 0.09 log CFU/g) compared to the control and HFHS groups (6.97 ± 0.07 and 7.01 ± 0.17 log CFU/g). A notable increase was also observed in the total viable count of anaerobic bacteria in the fecal samples of functional food fed mice (7.77 ± 0.28 log CFU/g). Consumption of the functional food led to a substantial increase in the levels of these beneficial bacteria compared to both the control and high‐fat, high‐sugar diet groups. Specifically, the functional food group exhibited significantly higher counts of lactic acid bacteria (8.01 ± 0.15 log CFU/g) and bifidobacteria (7.61 ± 0.17log CFU/g) compared to the control (7.74 ± 0.32 log CFU/g and 6.85 ± 0.32 log CFU/g) and high‐fat high‐sugar (7.55 ± 0.30 log CFU/g and 6.87 ± 0.50 log CFU/g) diet groups. Overall, these findings suggest that the consumption of functional food positively modulates fecal microbiota composition by promoting the growth of beneficial bacteria, which may confer health benefits to the host.

**FIGURE 4:**
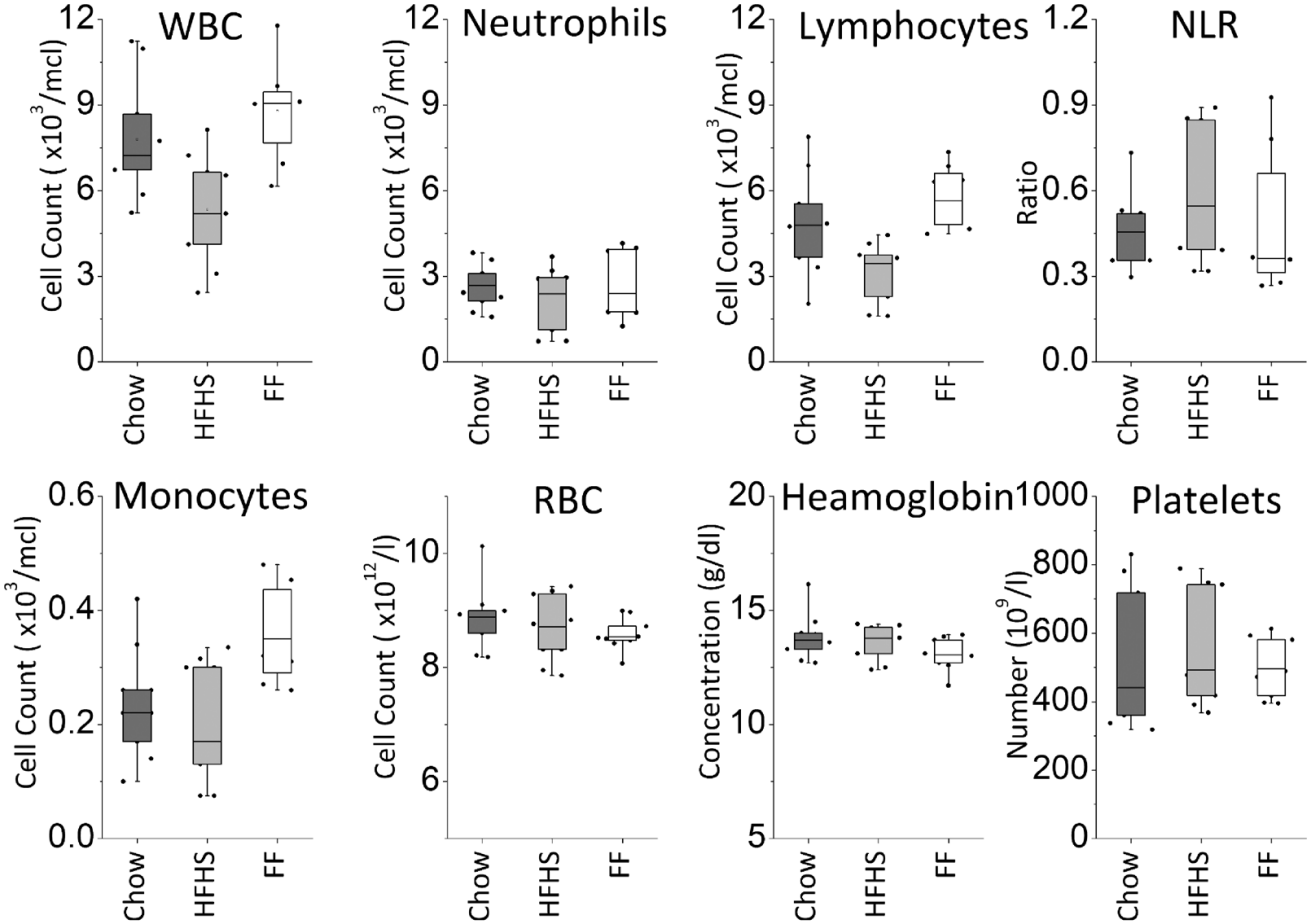
Hematological and immunological profiling in assessing the potential anti‐aging efficacy of cooked flatbread consumption: (a) White blood cell count (WBC) for assessing systemic immune function; (b) neutrophils count indicative of the innate immune response; (c) lymphocytes count indicative of adaptive immunity; (d) neutrophil‐to‐lymphocyte ratio (NLR), a systemic inflammatory marker, (e) monocytes count (f) red blood cell count (RBC), (g) hemoglobin content, (h) platelet count. The same lowercase letters indicate no statistically significant difference in the mean of the samples between mice fed the control diet, those fed the functional food, and those fed with HFHS diet.

Figure 4 represents the hematological parameters of mice samples on the 32nd day of the experiment. The median white blood cell (WBC) count of mice fed with functional food was measured at 9.07 × 10^3^/µL, which was statistically similar to that of chow‐fed mice (7.22 × 10^3^/µL, *p* = 0.328) but significantly higher than that of HFHS‐fed mice (5.19 × 10^3^/µL, *p* = 0.004). The increase in WBC count in functional food‐fed mice can primarily be attributed to a rise in lymphocyte count, estimated at 5.63 × 10^3^/µL, a value similar to that of chow‐fed mice (4.79 × 10^3^/µL, *p* = 0.594). In contrast, HFHS‐fed mice exhibited significantly lower lymphocyte counts (3.44 × 10^3^/µL, *p* = 0.001). Additionally, the monocyte count in functional food‐fed mice was noted to be 0.35 × 10^3^/µL, significantly higher than that of chow‐fed mice (0.22 × 10^3^/µL, *p* = 0.014) and HFHS‐fed mice (0.17 × 10^3^/µL, *p* = 0.016). However, no statistically significant difference (*p* > 0.05) was observed in neutrophil count across any of the groups, ranging between 2.38 to 2.67 × 10^3^/µL in mice fed with functional food.

Median red blood cell counts did not significantly differ within any of the groups, maintaining a range between 8.53 to 8.87 × 10^12^/L. Hemoglobin content showed a slight variation, with HFHS‐fed mice exhibiting a slightly higher level (13.37 g/dL) compared to functional food‐fed mice (13.05 g/dL) and chow‐fed mice (13.65 g/dL), although this difference was not statistically significant. Platelet count was found to range from 441 × 10^9^/L to 495 × 10^9^/L across all groups.

## 4 DISCUSSION

In recent years, there has been a growing interest in functional foods and their potential role in enhancing health and preventing diseases (Gagesch et al., 2023; Siró et al., 2008). Functional foods are defined as foods that provide additional health benefits beyond basic nutrition. These foods may be designed to promote health by addressing specific physiological needs. For a functional food to be recognized for its health‐promoting properties, it should demonstrate the ability to support some of the key aspects of health, such as but not limited to, reducing chronic inflammation, enhancing insulin sensitivity, managing the gut micro‐flora, and mitigating oxidative stress. Functional foods with high polyphenol content, omega‐3 fatty acids, vitamin D, and others bioactive components, can contribute to overall health by supporting metabolic balance, immune function, and cellular integrity, which may also retard the aging process (Luo et al., 2021; Hayes 2010).

With this aim, a premix for flatbreads with high protein content, rich in antioxidants, and serving as a potential source of omega acids was designed. The inclusion of pulses, millets, whey protein isolate, and seeds of flax, chia, and pumpkin was strategically chosen to maximize nutritional benefits. Pulses were selected for their high protein content, while millets contributed complex carbohydrates and polyphenols, enhancing the product's nutritional profile. Additionally, whey protein isolate served as a source of protein fortification, further boosting the protein content of the flatbread. Meanwhile, seeds such as flax, chia, and pumpkin act as natural sources of omega‐3 fatty acids (Petropoulos et al., 2021; Saini et al., 2021), adding to the product's health‐promoting properties (Elagizi et al., 2021; Saini et al., 2021). Nutritional analysis showed that the cooked flatbread made from the premix contained 10.7 g of protein, 2.2 g of fat, and 35 g of carbohydrates per 100 g of product. Notably, the product's protein content aligns with European Union standards for a protein‐rich designation, underscoring its potential as a valuable dietary component (European Commission's Food Safety website). The product also exhibited in vitro antioxidant activity in both the premix and the cooked product. It was noted that cooking enhanced the total phenolic content of the product but slightly reduced reducing power and radical scavenging activity. The increase in the detected phenolic concentration may be attributed to the enhanced extraction due to the matrix softening effect of the thermal processing (Gallardo‐Guerrero et al., 2008). Thermal processing may deteriorate the biological activity of the phytochemicals in food, thus resulting in increased measurable phenolic content but lower antioxidant activities (Putriani et al., 2022; Sablani et al., 2010). Most existing premixes with millets and pulses focus on fiber and micronutrients rather than protein enrichment. Because most lacto‐vegetarian diets in India are low in protein, the premix was developed with the aim of achieving ∼21% protein content while providing a balanced nutritional profile. The inclusion of whey protein isolate further boosts essential amino acid content, making it superior for muscle repair and overall nutrition. Also, the premix is rich in phenolic compounds and retains notable DPPH and hydroxyl radical scavenging activity after cooking, which means it may actively help combat oxidative stress. Most millet‐ or pulse‐based premixes lack data on how cooking impacts antioxidant properties.

The rheological analysis of the premix revealed complex interactions at interplay between the starch, non‐starch carbohydrates, fibres, protein, and fats in it. The most notable observations are the presence of two peaks during the cooling phase of the pasting analysis. The hydration, swelling, and gelatinizaton of the starch resulted in an initial rise in viscosity, but the staged gelation and aggregation of whey protein isolate, which is the major source of protein in the premix, may result in the increased viscosity during cooling. Similar high viscosity peaks were observed in the ternary system of wheat starch‐oleic acid‐gluten, which were attributed by Du et al., 2023 to the re‐association and entanglement of gelatinized wheat starch with the fatty acid and protein resulting in complex formations. The presence of whey protein isolate and other proteins from millets and pulses and fats are likely to cause the increase in viscosity of the premix suspension during the cooling. Zhang and Hamaker (2005) have reported the increase in the viscosity during cooling upon the addition of free fatty acid in defatted sorghum flours and also in flours of rice and corn. In fact, the interaction of protein with fatty acid was shown to be responsible for this rise in viscosity. Starch retrogradation, possibly occurring in distinct phases due to the starch from diverse sources and the modulating effects of other components in the premix, could also contribute to the viscosity increase. Only chia flour rich in gum, mucilage and protein is reported to have increased viscosity at 80–90°C but cooling resulted in syneresis and lower viscosity (García‐Salcedo, 2018), thus the incorporation of fiber‐rich chia and flax seeds may not result in the observed phenomenon. Furthermore, the hydration and potential gelation of soluble fibers, alongside the crystallization of fats from the powdered oilseeds, may contribute to the observed peaks. These phenomena, acting synergistically, likely result in the unique biphasic viscosity increase during cooling and highlight the intricate structural changes occurring within the premix as temperature decreases.

The assessment of the impact and efficacy of functional foods on general health is paramount in ensuring their suitability for long‐term consumption (Granato et al., 2020). In the present study, the functional food was assessed by monitoring its impact on the overall health of the subjects, which included the weight gain in Balb/c mice, estimation of antioxidant status of their splenocytes, and examining immune cell frequencies in the spleen. Throughout the duration of the study, both the test group mice fed with functional food and the control group fed with standard chow diet maintained similar weight gain trajectories, suggesting that the functional food did not hinder normal growth or metabolic health. While weight gain is commonly associated with over‐nutrition and obesity‐related complications, it is also essential for the normal growth and development of mice, suggesting sound metabolic health and nutritional status.

The measurement of total reactive oxygen species (ROS), mitochondrial ROS, and glutathione (GSH) levels provides valuable insights into cellular oxidative stress and antioxidant defense mechanisms (Khor et al., 2017; Truong et al., 2018). Our analysis revealed no significant differences in the antioxidant status of splenocytes between the test group consuming the functional food and the control group fed a standard diet, implying that the functional food did not induce oxidative stress or interfere with antioxidant defense mechanisms. This is a crucial finding, as oxidative stress is implicated in various pathological processes and is closely linked to aging and age‐related diseases (Ames et al., 1993; Lee et al., 2023). Furthermore, the evaluation of immune cell populations in the spleen revealed comparable levels of CD4+ cells, suggesting that the functional food might not alter T cell‐mediated immune responses significantly. However, the reduction in CD11b+, CD19+, CD14+, and CD16/32+ cells warrants further investigation. These cell populations play diverse roles in innate and adaptive immunity, including phagocytosis, antigen presentation, and antibody production (Zhao et al., 2009). In this context, the ability of functional food to maintain or modulate immune cell populations without inducing oxidative stress is promising. These findings are highly promising as they suggest that the functional food is not only safe for consumption but also exerts no adverse effects on cellular oxidative balance, and may support immune health while potentially mitigating immune decline caused due to age‐related and chronic diseases like diabetes mellitus, chronic kidney disorders, autoimmune disorder and lifestyle related factors including stress, depression, poor sleep, alcohol consumption and smoking disorders.

This study also compared the physiological outcomes of feeding mice with functional food versus a high‐fat, high‐sugar (HFHS) diet. Despite consuming equivalent quantities of food, mice across all dietary groups exhibited similar weight gain over the 32‐day experimental period, indicating that the functional food did not significantly impact weight gain compared to the standard chow or HFHS diets. Interestingly, the glucose levels in the serum of functional food‐fed mice were maintained similar to standard chow diet‐fed mice, but significantly raised in HFHS‐fed mice. The maintenance of glucose levels in functional food fed mice is significant because dys‐regulated glucose metabolism is closely linked to metabolic aging and associated diseases like type 2 diabetes. On the contrary, elevation in serum glucose levels observed in mice fed the HFHS diet raises concerns about its detrimental effects on metabolic homeostasis. This observation aligns with existing literature linking high‐fat, high‐sugar diets to metabolic dysregulation and increased risk of age‐related diseases such as diabetes and cardiovascular disorders (Alzoubi et al., 2013; Dominguez and Barbagallo, 2016; Heinonen et al., 2014; Panchal et al., 2011). The lack of significant differences in weight gain among the dietary groups suggests that factors other than caloric intake may be influencing metabolic outcomes (Heinonen et al., 2014). Excessive consumption of high‐calorie, low‐nutrient foods can lead to insulin resistance, inflammation, and oxidative stress, accelerating the aging process and increasing the risk of chronic diseases such as diabetes and cardiovascular disorders. The functional food may have properties that mitigate metabolic disturbances, although its impact on weight regulation in this short‐term study was not apparent.

The study also indicates improvements in white blood cell, lymphocyte, and monocyte counts, suggesting enhanced immune function by the functional food. This may contribute to resilience against infections, chronic and lifestyle disorders, and age‐related diseases. The similarity of NLR in control mice and functional food fed mice further indicates a dampened inflammatory response compared to HFHS fed mice, suggesting the potential affect of functional food on mitigating the chronic low‐grade inflammation associated with various chronic diseases and also age‐related ‘inflammaging’. By modulating immune function, inflammation, and hematopoiesis, functional food may offer a multifaceted approach to addressing physiological decline and improving overall health and well‐being.

Also, the microbial composition of mice fecal samples revealed significant differences in the fecal microbiota of mice fed on functional food compared to those fed on standard chow or a high‐fat, high‐sugar diet. A notable increase in the total viable count, lactic acid bacteria, and bifidobacterium in the fecal samples of mice fed on functional food suggests a prebiotic effect of the functional food. Lactic acid bacteria and bifidobacterium are well‐known probiotics with various health‐promoting properties, including modulation of immune responses and enhancement of gut barrier function (Ashaolu, 2020). Bifidobacteria are known for producing short‐chain fatty acids (SCFAs) like butyrate, which have anti‐inflammatory properties and contribute to gut health. It is known that the gut microbiome is essential for immune function and metabolic regulation, and age‐related dysbiosis can lead to chronic inflammation. The functional food flatbread premix might act as a source of dietary fibers that resist digestion in the upper gastrointestinal tract and reach the colon, serving as fermentable substrates for beneficial bacteria. Sorghum and pearl millet are high in resistant starch like arabinoxylans, green gram and chickpea are rich in galactooligosaccharides (GOS), seeds of pumpkin, flax and chia are rich in soluble fibers like mucilage and also insoluble fibers (Chen et al., 2019; Schupfer et al., 2021; Wang et al., 2022; Moczkowska et al., 2019; Tamargo et al., 2020). This increased fiber and slow‐fermenting carbohydrates can modulate the overall microbial load by providing energy sources for both aerobic and anaerobic bacteria. Flax seeds contain oligosaccharides which enhance Bifidobacterium populations (Livingston et al., 2023), while chickpeas and green gram, rich in raffinose family oligosaccharides, enhance Lactobacillus and Bifidobacterium populations (Amorim et al., 2020; Elango et al., 2022). Millets contain bound phenolics compounds, which can positively modulate the gut microbiota (Loo et al., 2020; Rocchetti et al., 2022). Fermentation of these prebiotic fibers by gut bacteria results in the production of SCFAs which create a more acidic environment in the gut, lowering the pH which selectively inhibits pathogenic bacteria while promoting the growth of beneficial bacteria like Lactobacillus and Bifidobacterium. The bioactive polyphenols also have antimicrobial properties against harmful bacteria while serving as substrates for beneficial gut microbes (Aravind et al., 2021; Rodríguez‐Daza et al., 2021).

Lignan from flax seeds, various flavonoids, phenolic acids, and tannins from millets, pulses and pumpkin seeds, vitexin and isovitexin from green gram act as microbiota modulators and improve the gut microbiota balance. Pulses like chickpeas and green gram provide fermentable plant proteins, which can promote the production of beneficial metabolites when metabolized by gut bacteria. Amino acids such as arginine and glutamine in these ingredients can support gut epithelial integrity and promote beneficial bacteria. Seeds of pumpkin, flax, and chia seeds are rich in alpha‐linolenic acid (ALA), a plant‐based omega‐3 fatty acid which have been shown to modulate gut microbiota by promoting Lactobacillus and Bifidobacterium, while reducing inflammation‐associated bacteria. By acting similar to prebiotics, the functional food may help sustain gut health and overall systemic health. Notably, the HFHS diet led to worse outcomes in these parameters, underscoring the protective effects of the functional food. These findings collectively suggest that the functional food supports health by maintaining key markers of metabolic and immune health, protecting against oxidative stress, thereby potentially extending health span. Unlike many studies that rely only on in vitro tests, this premix was tested in Balb/c mice, confirming no adverse oxidative or immune effects. It promoted a healthy gut microbiota, a critical feature not commonly evaluated in standard premixes. Enhanced WBC and lymphocyte counts suggest an immune‐boosting effect, which is rarely studied in functional food formulations.

## 5 LIMITATIONS AND FUTURE SCOPE

While this study describes the development of a nutritionally adequate, ‘complete meal’ and provides valuable insights into its potential benefits of mitigating metabolic disturbances, further research is warranted to elucidate the underlying mechanisms. Also, various parameters of the food product, like the texture of the food and storage stability of the premix, consumer acceptance, and environmental impact, were not evaluated. Also, the product developed was not entirely plant‐based, and financial affordability in terms of daily consumption was not addressed. The short duration of the experimental period and the focus on the use of traditional techniques for microbial characterization of gut microbiota or estimation of hematological parameters is an important limitations of the present study.

Future research should explore additional parameters such as biochemical markers, organ function, and long‐term effects to provide a more comprehensive assessment of the impact and efficacy of the functional food product.

## 6 CONCLUSION

In conclusion, this study describes the development of a nutritionally adequate functional food and its potential role in promoting health. Through meticulous evaluation, a premix has been formulated that can enhance health benefits and cater to the growing demand for nutritious and functional dietary options in modern society. This study also provides evidence supporting the potential of the functional food product to maintain metabolic health. The comparable weight gain, unaltered antioxidant status of splenocytes, and positive changes in hematological parameters and fecal microbiota in mice emphasize the consumption of nutrient‐dense foods over calorie‐dense, low‐nutrient options to support overall well‐being and longevity. Further research in this area holds promise for developing functional food variants that are nutritionally adequate and rich in bioactive compounds that may combat metabolic disorders and improve quality of life.

## AUTHOR CONTRIBUTIONS

**Shraddha A. Bhoir**: Methodology; conceptualization; investigation; visualization; resources; data curation; writing—original draft. **Deepak Sharma**: Methodology; software; resources; writing—review and editing; validation; supervision; investigation. **Sahayog Jamdar**: Conceptualization; methodology; supervision; resources; writing—review and editing; validation.

## CONFLICT OF INTEREST STATEMENT

The authors declare no conflicts of interest.

## Supporting information



Supplementary Table 1.: Composition of some representative market samples of similar flatbreads and its proximate analysis.
