# Supplementary material for: Millets, pulses, and oil seeds‐based flatbread premix: A protein‐rich functional food for healthier dietary habits and prevention of lifestyle disorders
Source: J Food Sci. 2025 Apr 24;90(4):e70209. doi: 10.1111/1750-3841.70209 (PMC12019912; doi:10.1111/1750-3841.70209)
Supplement: Supplementary file 1 — Supplementary Table 1.: Composition of some representative market samples of similar flatbreads and its proximate analysis. [file JFDS-90-0-s001.docx]

Supplementary Table 1.: Composition of some representative market samples of similar flatbreads and its proximate analysis.

|  | Market Sample 1 | Market Sample 2 | Market Sample 3 | Market Sample 4 |
| --- | --- | --- | --- | --- |
| **Millets** |  |  |  |  |
| Jowar flour | 29.7 | ✓ | 10 | ✓ |
| Bajra flour |  | ✓ | 10 |  |
| Banyard Millet |  |  | 10 |  |
| Little Millet |  |  | 10 | ✓ |
| Foxtail Millet |  |  | 10 |  |
| Finger Millet |  |  |  | ✓ |
| **Pulses** |  |  |  |  |
| Sprouted moong flour | 29.7 | ✓ | 50 |  |
| bengal gram flour |  | ✓ |  | 27 |
| masur dal flour |  | ✓ |  |  |
| urad dal flour |  | ✓ |  |  |
| whole green gram flour |  | ✓ |  |  |
| pegion pea flour |  | ✓ |  |  |
| moth bean |  | ✓ |  |  |
| **Cereals** |  |  |  |  |
| Rice flour /Flakes |  | ✓ |  | ✓ |
| Wheat flour/ semolina/ refined flour |  | ✓ |  | ✓ |
| oats flour |  |  |  |  |
| Corn flour |  |  |  | ✓ |
| **Other ingredients** |  |  |  |  |
| flax seeds |  |  |  |  |
| jackfruit flour |  |  |  |  |
| **Proximate Analysis** |  |  |  |  |
| Energy (kCal) | 368 | 390 | 382.11 | 380 |
| Protein (g) (energy from protein) | 13.7  (54.8 kCal, 14.9 % of total energy) | 8  (32 kCal, 8.2 % of total energy) | 12.31  (49.24 kCal, 12.9 % of total energy) | 14  (56 kCal, 14.7 % of total energy) |
| Carbohydrates (g) | 71.9 | 78 | 73.79 | 70 |
| Fats (g) | 2 | 5.3 | 4.19 | 4.5 |
